# STING agonists as promising vaccine adjuvants to boost immunogenicity against SARS-related coronavirus derived infection: possible role of autophagy

**DOI:** 10.1186/s12964-024-01680-0

**Published:** 2024-06-03

**Authors:** Aysa Rezabakhsh, M. Reza Sadaie, Alireza Ala, Yousef Roosta, Solomon Habtemariam, Adeleh Sahebnasagh, Mohammad Rafi Khezri

**Affiliations:** 1 Cardiovascular Research Center, Tabriz University of Medical Sciences, Tabriz, Iran; 2 NovoMed Consulting, Biomedical Sciences, Germantown, Maryland USA; 3 Emergency and Trauma Care Research Center, Tabriz University of Medical Sciences, Tabriz, Iran; 4 Hematology, Immune Cell Therapy, and Stem Cells Transplantation Research Center, Clinical Research Institute, Urmia University of Medical Sciences, Urmia, Iran; 5 Pharmacognosy Research and Herbal Analysis Services UK, University of Greenwich, Kent, UK; 6 Clinical Research Center, Department of Internal Medicine, North Khorasan University of Medical Sciences, Bojnurd, Iran; 7 Reproductive Health Research Center, Clinical Research Institute, Urmia University of Medical Sciences, Urmia, 5715799313 Iran

**Keywords:** Autophagy, COVID-19, Immunogenicity, SARS-CoV-2, Stimulator of interferon gene, Vaccine adjuvant

## Abstract

As a major component of innate immunity and a positive regulator of interferons, the Stimulator of interferon gene (STING) has an immunotherapy potential to govern a variety of infectious diseases. Despite the recent advances regarding vaccines against COVID-19, nontoxic novel adjuvants with the potential to enhance vaccine efficacy are urgently desired. In this connection, it has been well-documented that STING agonists are applied to combat COVID-19. This approach is of major significance for boosting immune responses most likely through an autophagy-dependent manner in susceptible individuals against infection induced by severe acute respiratory syndrome Coronavirus (SARS‑CoV‑2). Given that STING agonists exert substantial immunomodulatory impacts under a wide array of pathologic conditions, these agents could be considered novel adjuvants for enhancing immunogenicity against the SARS-related coronavirus. Here, we intend to discuss the recent advances in STING agonists’ recruitment to boost innate immune responses upon vaccination against SARS-related coronavirus infections. In light of the primordial role of autophagy modulation, the potential of being an antiviral vaccine adjuvant was also explored.

## Introduction

The stimulator of interferon genes (STINGs), encoding the transmembrane protein 173, plays a critical role in innate immunity instigation against a wide variety of infections [1–3]. Recently, the STING pathway has been proposed as a cancer vaccine adjuvant primarily due to the fact that endogenous activation of STING results in the modulation of cellular immunity mainly mediated by cytotoxic CD8^+^ T cells [4]. In coronavirus infection, stimulation of the innate and adaptive immune systems is mediated by interferons (IFNs), containing IFN-α and –β subunits [5]. Of note, both IFN-I and -III are defined as cytokines that prompt the first-line defense against pathogens, particularly viruses [6]. Similar to other viruses, the severe acute respiratory syndrome coronavirus 2 (SARS‑CoV‑2) recruits advanced mechanisms for evading both host immune response and antiviral functions mediated by IFN-I and –III at multiple stages [7]. However, the potential clinical utility of STING agonists warrants further investigation.

Calling attention, activation of STING concurrently stimulates a multifaceted IFN-I-mediated immune response that further promotes the maturation and migration of dendritic cells (DCs), primes cytotoxic T cells, and natural killer (NK) cells for spontaneous immune responses [8]. Presumably, either persistent or aberrant activity of STING can suppress the immune response by engaging regulatory T cells (Tregs), infiltration of myeloid-derived suppressor cells (MDSC), and upregulation of the Programmed Cell Death 1 (PDCD1) gene [9, 10]. This can further restrict the antigen-presenting function of DCs through an indoleamine 2, 3-dioxygenase (IDO)-kynurenine-dependent immune tolerance and apoptosis induction [11, 12]. In COVID-19, IFN-I dysregulation is supposedly critical in disease pathogenesis [13]. In support of this hypothesis, either IFN-I pre-treatment or administration at the onset of the disease effectively prevents COVID-19 progression toward the severe form due to the autonomous antiviral state by diminishing viral load [14]. Regardless of autoimmune reactions and genetic disorders, IFN-I’s aberrance progressively promotes immunopathology, which can develop the severe form of COVID-19 [13]. Here, we aimed to discuss STING agonists’ and regulated autophagy effects in modulating immune responses upon the SARS-related coronavirus infections and COVID-19 vaccination.

## Published Research and Data interpretations

### STING-related mechanism of action

At the initial stage of the STING pathway, the cyclic guanosine monophosphate-adenosine monophosphate (cyclic GMP-AMP, cGAMP) synthase (cGAS) enzyme can sense cytosolic nucleic acid contents as a danger signal to instigate the STING-dependent IFN immune response [15, 16]. A proof-of-concept study demonstrated that viral infections induced by DNA and RNA viruses exhibit two distinct STING pathways [17], as follows:

First, upon binding to cytosolic DNA, cGAS increases the levels of 2’ 3’-cGAMP and canonical cyclic dinucleotide (CDN) [18], two secondary messengers synthesized from adenosine triphosphates (ATP) and guanosine triphosphates (GTP), in turn, promotes STING activation in the endoplasmic reticulum (ER) during a length-dependent manner. In better words, the IFN immune response in the presence of cytosolic DNA can emerge at the short lengths of DNA, containing at least 20–40 base pairs (bp), which is entirely dependent on cGAS irrespective of DNA and its broad length span, ranging from the least stimulatory length to several kilobases [19].

**Fig. 1:**
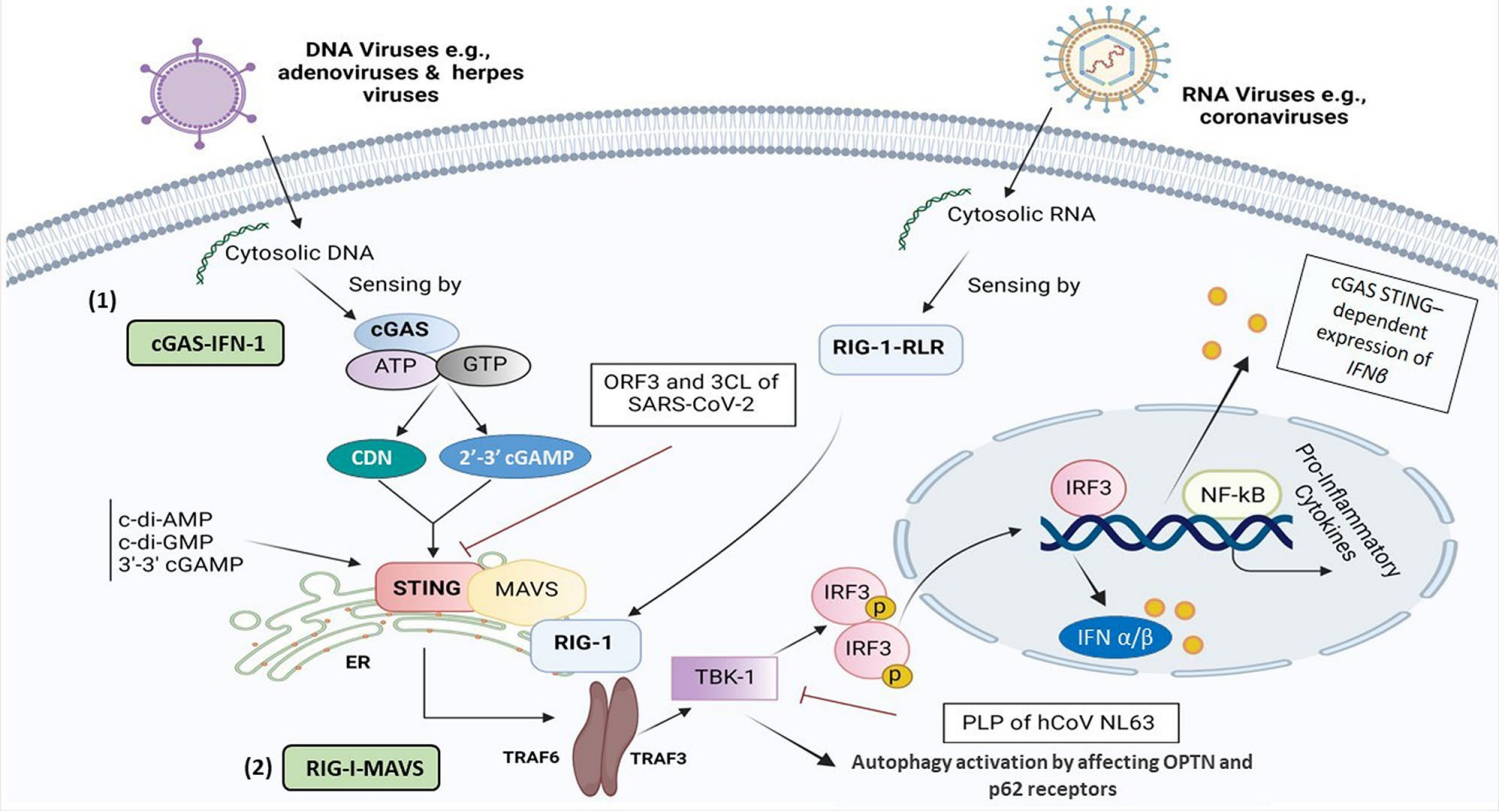
A schematic representation of STING-related cGAS and RIG-1-dependent signaling pathways in the presence of both DNA and RNA-based viruses, respectively. The drawing illustrates a comparison and shared molecular interplays and mechanisms involved in the stimulation and suppression of the immune system, in particular SARS-CoV-2 influence in the induction of inflammation and cytopathic effects through RIG-I, MAVS, TRIF, TBK1, TRAF3/6, and IRF3 signaling axis. This figure was created with BioRender.com. *Abbreviations* CDN, cyclic dinucleotide; cGAS, cyclic guanosine monophosphate-adenosine monophosphate (GMP-AMP) synthase; ER, endoplasmic reticulum; IFN-I, type I interferon, IRF3; interferon regulatory factor 3; MAVS, mitochondrial antiviral-signaling protein; NFκB, nuclear factor-κB; RIG-I, retinoic Acid-inducible gene I; RLR, RIG-like receptors; STING, stimulator of interferon response cGAMP; TBK1, TANK-binding kinase 1; TRAF3/6, Tumor necrosis factor receptor (TNFR) associated factors 3/6

The TANK-binding kinase 1 (TBK1) then activates IFNα/β by phosphorylation of IFN regulatory factor 3 (IRF3), a key effector of STING downstream, and non-canonical nuclear factor kappa-light-chain-enhancer of activated B cells (NFκB) [20, 21]. Ultimately, activated IFN-β mainly released from Plasmacytoid DCs, T lymphocytes (Th1, CD^4+^ T cells), and NK cells emerge in the target sites to promote the desirable antiviral effects (Fig. 1).

Second, in comparison to DNA viruses that STING deploys desirable effects through a cGAS-IFN-I dependent manner, STING could also hamper replication of the RNA virus at the translation, but not transcription level, to prevent viral protein synthesis. This appears to occur through the synthetic retinoic acid-inducible gene I (RIG-I) pathway, demonstrated using transfection of RIG-like receptors (RLRs) ligands, and antiviral gene expression stimuli [17]. In addition, STING and mitochondrial antiviral-signaling protein (MAVS) can coordinate their gene expression as co-regulators through positive feedback [22]. Mechanistically, there is a cross-link between RIG-I-MAVS and cGAS-STING of RNA and DNA sensing pathways, respectively, for more improvement of the evolutionarily conserved innate immune responses (Fig. 1). As illustrated in Fig. 1, the human coronavirus (hCoV) NL63, through its papain-like protease (PLP) domain, can interrupt STING-TBK1 interaction [23], leading to the suppression of IFNβ production. PLP impedes STING dimerization, K63-linked polyubiquitination of IFN-I, and negatively regulates IRF3 activation via interaction with STING- TNF receptor-associated factor (TRAF3)-TBK1 complex [24, 25]. TRAF6 is a crucial adaptor protein related to the interleukin (IL)-1 receptor/ toll-like receptor (TLR) family and TNF-receptor superfamily [26]. Both TRAF3 and − 6 are predominately involved in virus-triggered signaling by connecting upstream adaptor proteins to downstream protein kinases and transcription factors [27].

Whether or not PLP in the SARS-CoV-2 virus can affect the STING-TRAF3-TBK1 complex is yet to be determined.

### The cGAS-STING pathway in DNA/RNA-based viruses

Unlike SARS-CoV-2, other coronavirus infections induced by human coronavirus OC43 (HCoV-OC43) could not stimulate the STING pathway, while pharmacological stimulation of the STING-IRF3 pathway substantially hindered the HCoV-OC43 infection [28]. In this regard, diABZI exerted a desirable anti-coronavirus activity against both HCoV-OC43 and SARS-CoV-2 strains [28]. In contrast, other RNA viruses, such as flaviviruses exhibit a distinct characteristic of suppressing IFN-I production using NS2B/3 protease activity and cleavage of STING at residues 93–96 (LRRG) [29]. Furthermore, some enveloped RNA viruses, such as influenza A, can also instigate the STING-IFN axis independent of cGAS as a primary target for viral hemagglutinin glycoprotein or NS2B3 protease [30]. Also, infection by West Nile virus, an ssRNA virus, leads to higher viral loads and mortality rates in cGAS knockout mice. This was attributed to cGAS-mediated modulation of STING in the absence of direct sensing of the pathogen nucleic acid by cGAS [31]. In addition, some negative-strand RNA paramyxoviruses could trigger the cGAS-STING pathway. The last findings demonstrate that silencing the cGAS or STING gene reduced IFN-I production with a simultaneous enhanced paramyxoviral infection in vivo [32]. These observations advocate the feasibility assessments of developing virus-specific STNG adjuvants.

The outcomes of a comprehensive review have also corroborated that the RIG-I-MAVS pathway is a key cytosolic pathogen recognition receptor (PRR) in combating RNA viruses. The findings of this study highlighted that STING-RIG-1 agonists are taken into account as effective antiviral agents besides being vaccine adjuvants [22]. In parallel to these findings, other studies have also demonstrated the potent antiviral effect of STING induction through a RIG-1 agonist, namely 5’ triphosphorylated RNA (5’ pppRNA), which can increase STING induction both at transcriptional and translational levels following herpes simplex virus 1 (HSV), an enveloped dsDNA virus, infection [33]. In contrast to RNA, hepatitis B virus (HBV) DNA is also able to stimulate the innate immune response mediated by the cGAS–STING pathway. In addition, a sufficient amount of DNA transfected to hepatocytes in culture demonstrated reduced levels of DNA sensors compared to myeloid immune cells, resulting in HBV evasion of cGAS–STING sensing [34].

It can be also postulated that the STING plays a core effector role in priming downstream cascades in the interplay between distinct DNA and RNA sensing signaling pathways. Inhibition of the RIG-1-RLR-STING axis induced by evolving viral mechanisms should also be considered in host innate immune system failure, particularly in SARS-CoV-2 infection. Based on the results of a recent study, STING inhibition can be achieved through two distinct pathways:

1) A viral accessory protein named open reading frame (ORF) can prevent nuclear accumulation of p65 and subsequently inhibit the NFκB signaling,

2) The inhibitory effect of 3 C-like protease (3CL), as one of the main proteases targeted for therapeutic COVID-19 antiviral intervention, on K63 ubiquitination and potent suppression of STING-mediated NF-κB signaling [35].

As the STING mode of action following RNA/DNA viral infection, recent literature reported the elevated levels of monocytes with CD16 positive transcription factor T-box expressed in T cells (TBET) and CD14 positive IRF1, as well as SARS-CoV-2-specific CD8 + T cells in the COVID-19 convalescent plasma. These findings further demonstrate the genomic changes (chromatin remodeling) observed in adaptive immune cells to derive trained immunity by engaging the STING downstream effectors, i.e., IRFs, following COVID-19 infection [36].

### STING agonists

To date, booster doses of COVID-19 vaccines are recommended to maintain the proper immune responses against the emerging variants of SARS-CoV-2 [37]. As a novel approach, it has been thought that STING agonists could be developed as adjuvants to optimize the immunogenicity and efficacy of the vaccines [38]. For instance, 2’3’- and 3’3’-cGAMP, as well-known ligands of STING, can prime the innate immune response during *de novo* antigen-specific CD8^+^ T cells and IFN-I induction against RNA-based viral infection [39]. It has also been revealed that the nanoparticles harboring 2′3′-cGAMP isoforms could elicit protective CD8^+^ T cell-based antiviral responses against human immunodeficiency viruses-1 (HIV-1). Besides vaccination, this novel approach is also applicable to immunotherapy and prophylactics [39]. Another study also reported that flaviviruses such as yellow fever virus and dengue virus, possessing non-structural protein NS4B, can co-localize with STING and inhibit the STING-RIG-I-dependent signaling pathway [29]. Therefore, RLRs are responsible for the exclusive detection of cytosolic ss/ds RNA in the course of virus infection to further stimulate innate immune responses.

Given that STING agonists have poor bioavailability, Jang et al. designed exoSTING as a novel and distinct drug delivery system for exclusively delivering CDN into the target sites. This approach of engineered extracellular vesicles, extracted from HEK293 cells, is loaded with STING agonists, which simultaneously augments the CDN potency and the CD8^+^ T cells response. In addition, the wide therapeutic window of exoSTING also allows effective dose optimization [40]. Although the cGAS-STING pathway is well-established and elucidated in viral DNA sensing, it can also exhibit paramount functions in host innate immunity against distinct positive-sense single-stranded (+ ss) RNA viruses (e.g., human flaviviruses and coronavirus) with no DNA involvement in their life cycle [29]. The list and characteristics of various STING agonists are summarized in Table 1.

**Table 1:** The characteristics of various native and synthetic STING agonists

No.	Agonist Name	Year	Type	hSTING	Activity	Ref.
**1**	DMXAA	2013, 2019, 2020	Synthetic	No	Negatively targeting vasculature with the recruitment of type I IFN can propagate CD8 + T cell infiltration.	[1–3]
**2**	c-di-GMP and c-di-AMP	2019	Native	Yes	Cancer vaccine adjuvants	[4]
**3**	2’3’-cGAMP	2013	Native	Yes	A native agonist, following the interaction with STING, can facilitate apoptosis in HTLV-I-infected monocytes upon the IRF3-Bax complex production.	[5]
**4**	3’3’-cGAMP	2013	Native	Partial	More effective than DMXAA in activating the STING-IRF3-SATA1 axis	[5]
**5**	diABZI-4	2020, 2021	Synthetic	Yes	It has a potential therapeutic against respiratory viral infections induced by parainfluenza, rhinovirus, and SARS-CoV-2	[6, 7]

*Abbreviations* diABZI-4, diamidobenzimidazole; DMXAA, 5,6-dimethylxanthenone-4-acetic acid; GMP-AMP, guanosine monophosphate-adenosine monophosphate; IFN-I, type I interferon; IRF3, interferon regulatory factor; 3; hSTING, human STING; HTLV-I, human T-cell leukemia virus type one; MAVS, mitochondrial antiviral-signaling protein; NFκB, nuclear factor-κB; SARS-CoV-2, severe acute respiratory syndrome coronavirus 2; STING, stimulator of interferon genes

### Definition of the autophagy

Autophagy, is defined as a tightly preserved cellular process involvedin the turnover of worn-out components, i.e., damaged organelles, aggregated/unfolded proteins, and pathogen particles, resulting in cellular homeostasis, survival, and regulation of cell functions under various stress conditions and viral infection [41, 42]. According to the degradation mechanism and delivery route toward the lysosomes, autophagy is classified into three types: macroautophagy (formally defined as autophagy), microautophagy, and chaperon-mediated autophagy (selective autophagy) [43]. Moreover, the autophagy process consists of some sequential stages, including initiation and membrane nucleation, double-membrane autophagosome formation, lysosomal fusion (autophagolysosome formation), and lysosome-dependent degradation [43]. To regulate autophagy flux, various autophagy-related genes (*Atgs*) and specific marker proteins such as ATG3, ATG7, microtubule-associated protein 1 A/1B-light chain 3 (LC3), Becline-1, and SQSTM1 (P62) mediate different stages of autophagy [44, 45].

Beyond the housekeeping activity, autophagy functions within the immune cells, e.g., T lymphocytes, to further modulate pro-inflammatory processes following a viral invasion [46, 47]. Besides, autophagy can contribute to antigen processing and presentation in dendritic cells (DCs) [48]. In this sense, autophagy has been considered a defensive machinery against viral infection via degrading the pathogens into autolysosomes. As defense machinery, autophagy can be induced to antagonize viral infections by conveying cargo (either cytoplasmic virions or viral components) to lysosomes for targeted degradation. Subsequently, this mechanism provokes the innate immune response, antigen presentation, and clearance of recognized pathogens. Not surprisingly, the supremacy of autophagy has been harnessed to boost the efficacy of vaccination and to date, several autophagy inducers have emerged benefits as vaccine adjuvants [49–51].

**Fig. 2:**
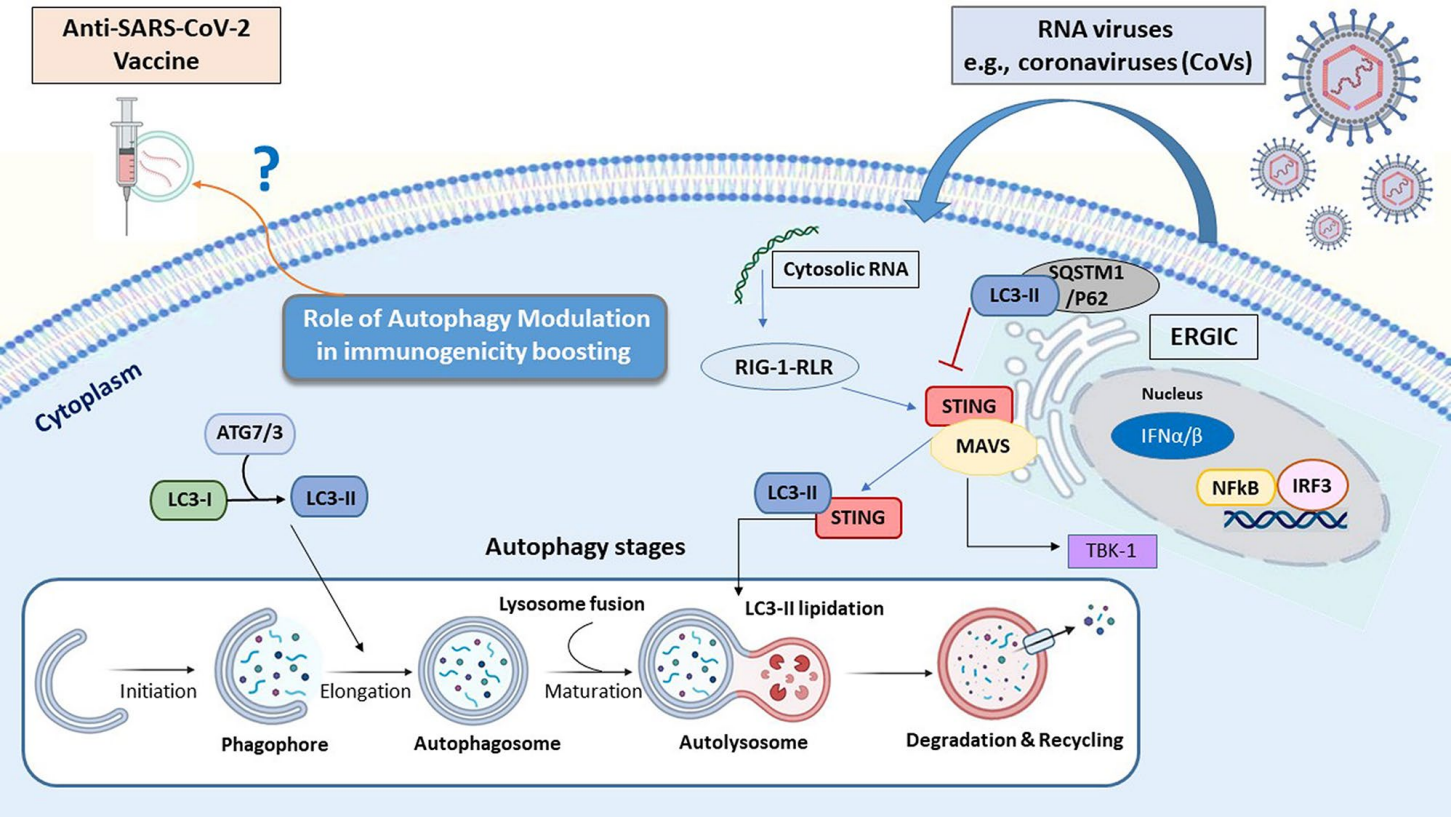
The interplay between autophagy flux and STING pathway following viral infection. Autophagy (macroautophagy) consists of four sequential stages: [1] initiation followed by membrane nucleation (to form phagophore) [2], phagosome formation and expansion (named elongation) [3], the lysosomal fusion to form autolysosome, and [4] final stage refers to the cargo degradation. The autophagy process is activated by numerous physiologic and pathologic stress conditions such as starvation, pathogen attacks. Following DNA/RNA viruses’ entry into the host cells both autophagy and STING pathways can activate to promote innate immune response. Beyond the interruption in lysosome fusion to promote LC3 lipidation induced by STING, some specific autophagy proteins such as LC3 and SQSTM1/P62 also block the STING-related pathway during different viral infections. *Abbreviation*: ATG, autophagy-related gene; ERGIC, ER–Golgi intermediate compartment; ER, endoplasmic reticulum; IFN-I, type I interferon, IRF3; interferon regulatory factor 3; LC3, Microtubule-associated protein 1 A/1B-light chain 3; STING, stimulator of interferon response cGAMP; NFκB, nuclear factor-κB

In this connection, several proteins involved in IFN-related signaling pathways are associated with autophagy regulation. For instance, the cGAS–STING pathway can trigger the autophagy flux following dsDNA sensing. As shown in Fig. 2, upon activation RIG-1-MAVS axis, STING transfers from the ER to the Golgi apparatus through the ER–Golgi intermediate compartment (ERGIC) [52], where STING triggers autophagosome formation by serving as a membrane source for LC3 lipidation (Fig. 2) [53].

### Antiviral impact of STING Signaling mediated by Autophagy

As a signaling adaptor protein, STING directly binds to cyclic di-GMP (c-di-GMP) and orchestrates pro-inflammatory cytokines secretion [54]. Released IFN-I is able to target multiple viral infections through a STAT-dependent manner and selective autophagy induction to eliminate pathogen particles [55]. Therefore, STING not only can stimulate the immune response, but it can also promote autophagy activity in a non-cGAMP (non-immune) manner to tune immune responses following viral infections [56]. In this regard, the STING pathway affects the autophagy flux through the activation of related receptors optineurin (OPTN) and adaptor protein p62 (SQSTM) in a TBK1-dependent manner [57, 58].

Available evidence indicated that African swine fever virus (ASFV), SARS-CoV-2, and porcine circovirus type 2 (PCV2) might regulate autophagy stages. For instance, the SARS-CoV-2 ORF7a protein activates the autophagy process via the AKT-mTOR-ULK1 pathway [43]. Following Zika virus (ZIKV) infection, STING also triggers autophagy by converting LC3-I into LC3-II [43]. Some viruses evade autophagy by targeting autophagolysosome formation in host cells. During SARS-CoV-2 replication, a viral protein named ORF7a hinders vesicular trafficking and subsequent fusion of autophagosome vesicles to and lysosomes by activating caspase 3 to degrade synaptosomal-associated protein 29 (SNAP29) [43]. For instance, Epstein–Barr virus (EBV) and Kaposi’s sarcoma-associated herpesvirus inhibit autophagy flux by selectively antagonizing the receptor SQSTM1/p62 [43].

#### The role of ORF Protein in Autophagy Flux

Recent literature also demonstrated that SARS-CoV-2-related ORF3a enables to interruption of the fusion of autophagosomes to the lysosomes through binding with a homotypic fusion and protein sorting (HOPS) component, VPS39, in the lysosome site. This simultaneously blocks autophagy flux via cessation of the STX17-SNAP29-VAMP8 SNARE assembly to evade viral degradation into the autophagosomal-lysosomal compartment [59, 60]. In addition, it has been well-established that SARS-CoV-2 ORF9b negatively governs STING-mediated antiviral immunity and thus accelerates viral replication. In this respect, inhibition of IFN-I and -III induced immune response mediated by SARS-CoV-2 ORF9b can emerge through blocking RIG-I, MAVS, TRIF, TBK1, and IKKε signaling axis [61].

Upon screening twenty-nine SARS-CoV-2-encoded viral proteins, Han et al. also recognized that SARS-CoV-2-related ORF10 overexpression has the potential to antagonize cGAS–STING signaling and STING-mediated innate antiviral immunity [62]. In detail, ORF10 impairs the STING–TBK1 association, blunts ER-to-Golgi trafficking of STING, and ultimately impedes STING-induced autophagy [62].

Overall, adjuvants promoting the STING-related pathways may shift the immune response in favor of a robust protective antiviral neutralization. These novel strategies and technologies should mitigate the pathological outcomes of SARS-CoV-2-related infections and relapses, by activating the autophagy and STING-mediated antiviral efficacy of viral vaccines. In Fig. 2 depicts sensing viral cytosolic RNA pathways, the interplay between autophagy flux and STING agonists.

This evidence further suggests that employing STING agonists, as vaccine adjuvants, may augment the efficacy of the SARS-CoV-2 vaccine and variants of concern.

#### Conflicting effects of Autophagy in SARS-CoV-2 infection

Numerous studies explored autophagy (virophagy) modulation and its possible effect on SARS-CoV-2 infection [63]. Since autophagy influences viral cell entry, transcription, and translation, two scenarios have been represented based on the dual function of autophagy:

 [1] Antiviral effect (regulated autophagy): disrupting the viral replication cycle followed by sequestration of viral particles and subsequent encompassing through autophagosomes and final degradation by lysosomes’ hydrolytic enzymes.

 [2] Pre-viral effect (dysregulated autophagy): following the blocking of the autolysosome formation via viral particles most likely through ORF3a protein, the viral proteins are released from the host cells to the extracellular space [64].

Noticeably, a prominent role of autophagy in the inflammatory response (especially thrombotic immune-inflammatory syndrome) follows SARS-CoV-2 infection [65]. Autophagy induction could impede the consecutive robust inflammatory response triggered by SARS-CoV-2 infection, leading to a multi-organ failure [65]. Calling attention, cells exposed to the SARS-CoV-2 infection can escape immune response mediated by IFN-I induction (markedly increase of green fluorescent protein (GFP)-LC3 positive autophagosomes) in vitro [66]. Consequently, blocking the excessive autophagy flux using selective inhibitors, like 3-methyadenin (3-MA) and non-selective inhibitor chloroquine leads to the inhibition of viral replication and the reduction of viral load [44, 45]. However, another study revealed that increased autophagy flux using the mammalian target of rapamycin (mTOR) inhibitors, e.g., metformin can exert an antiviral effect through the PI3K-AKT-mTOR axis, and hinder the interaction of SARS-CoV-2 proteins, including non-structural protein and ORFs with mTORC1, La-related protein 1 (LARP1), and 4E-BP in host cells [67].

Modulation of autophagy seems a promising target against SARS-CoV-2 spike protein (S-protein) related diseases to shed light on developing novel therapeutic platforms [68]. Recent research found that autophagy-induced peptide C5 using human adenovirus (HAd) vector-based vaccine results in better cell-mediated immune response against SARS-CoV-2 when compared to the HAd vector-based vaccine with S protein alone [69].

### The cGAS-STING pathway in the setting of COVID-19 severity

Regarding the COVID-19 pathogenesis, subsequent viral cytopathic effects, and host immunopathology, it has been well-established that the pneumocyte fusion ensues through cleavage of the S-protein via specific proteases at the S1/S2 and the S2’ sites [70]. After that, binding with the angiotensin-converting enzyme 2 (ACE2) receptor, in part activates the cGAS-STING pathway and related IFN-I response [71], which can contribute to worsening the COVID-19 severity [70]. Indeed, viral PLP mainly interacts with the STING pathway to block downstream IFN secretion in the early stage of the SARS-CoV-2 infection [72]. While in the late phase of infection, damaged DNA (i.e., oxidized mitochondrial DNA) potentially activates the cGAS-STING pathway as a result of the micronuclei and syncytia formation in the infected cells [73], leading to excessive release of IFN-β and substantial cytokine storm phenomenon during IRF-3 and NFκB activation [70, 72, 74]. In parallel with this, a recent study corroborated that the cGAS-STING pathway is a leading cause of aberrant IFN-I-induced immune responses in COVID-19, via mitochondrial DNA release in the endothelial cells, while pharmacological blocking of the STING pathway significantly reduced inflammatory status, particularly in the respiratory system [71]. Others advocate that designing STING inhibitors, such as H-151and VS-X4, could serve as novel therapeutic options in the late phase of SARS-CoV-2-like infections to alleviate the hyper-inflammation, as a result of the persistent STING activation, particularly in severe or critically ill patients [71, 75, 76].

Feasibilities of developing these technologies await controlled clinical investigations. However, as a safety issue, it should be pointed out that the detrimental effect of IFN (STING overstimulation) can potentially occur by enhancing the delayed innate immunity, followed by the excessive influx of pathogenic monocytes-macrophages with a worse prognosis [74].

### The impacts of delayed activation of the STING pathway in SARS-CoV-2

SARS-CoV-2 can induce a delayed innate immune response in respiratory epithelial cells, which likely leads to the virus establishment in the respiratory tract [77]. On this basis, both in vivo and in vitro studies highlighted that early treatments with STING agonists, e.g., diABZI, are promising therapeutic options to govern viral infection by limiting viral replication and inflammatory response in an IFN-dependent manner [77]. A more recent study also noted that the pharmacological antiviral activity of STING agonists (e.g., CDNs) toward the SARS-CoV-2 infection is highly appreciable [78]. In addition, the small-molecule STING agonist, diamidobenzimidazole (diABZI) compound, exhibited a desirable anti-SARS-CoV-2 effect against a broad array of variants, especially beta COVID-19 variant (B.1.351), by hampering the virus replication via an IFN-I independent manner under the experimental settings [78]. Interestingly, this small molecule can further mitigate the ability of SARS-CoV-2 to evade immunity via boosting IFN signaling and the TBK1/IRF-3 in primary human airway epithelial cultures [79].

In parallel with this finding, Humphries and colleagues also emphasized that either prophylactic or therapeutic administration of diABZI-4 on the onset of the disease completely restricted SARS-CoV-2 replication through transient pro-inflammatory cytokines production and myeloid and lymphocyte activation, particularly in the lung epithelial cells in both in vitro and in vivo settings and exerted a predominant antiviral effect [80]. Furthermore, they showed that intranasal administration of diABZI-4 in K18-ACE2-transgenic mice infected with SARS-CoV-2 can induce rapid short-lived activation of STING, as a host-directed therapy, through either IFN-dependent or IFN-independent manners in K18-ACE2-transgenic mice infected with SARS-CoV-2 [81].

In a clinical setting, delayed activation of the STING pathway, T cell delayed excessive responses, and delayed cytokine over-secretion were recently observed in patients with severe COVID-19, most likely due to DNA damages, highlighting the dichotomous role of STING in the case of SARS-CoV-2 [82]. To further ascertain the delayed activation of the STING pathway was demonstrated in an experimental model of SARS-CoV-2-induced hyperinflammatory immune response in the lung epithelial cells in culture [83]. Beyond the upregulation of inflammatory cytokines, the authors found the distinct activation of NF-κB and suppression of IRF3 in the infected cells [83]. Despite early-stage induction, type I IFNs can restrict SARS-CoV-2 infection. A persistent cGAM-STING-dependent type I IFN signature is mainly associated with excessive inflammation and subsequent adverse clinical outcomes [84]. Moreover, a lung-on-chip model demonstrated that the release of damaged mitochondrial DNA is involved in STING signaling-dependent type I IFN production and endothelial cell death, which could be reversed via pharmacological inhibition [84]. However, further assessment of STING polymorphism would be valuable toward better management of severe COVID-19.

### Applied STING agonists against SARS-CoV-2

Some synthetic adjuvants, including modified emulsions (such as Essai O/W 1,849,101, AS03, AS37, CpG1018 alum) and alum combined with SARS-CoV-2 spike protein receptor-binding domain nanoparticle (RBD–NP) are being studied in multiple ongoing clinical trials to augment the neutralizing-antibody responses [85]. Notably, the results of an experimental study revealed that the intranasal delivery of a cGAMP nanoparticle as an immune-antiviral agent, named NanoSTING, exerted a broad-spectrum antiviral property and profoundly elicited as a prophylactic and therapeutic aid against neutralization-resistant SARS-CoV-2 variants, e.g., Omicron. Noteworthy, it can also confer durable protection against variants with high prevalence, such as Alpha and Delta in the animal model [86].

### Advances in STING agonist-adjuvant vaccines against COVID-19

Although various platforms of vaccines were designed to combat SARS-CoV-2 infection, due to the limited potency of the available vaccines, and the propensity of coronaviruses to mutate can evade the highly protective immune response, thus precipitating an enduring infection and compromise the effectiveness of the vaccine distribution globally. The necessity of using strong adjuvants may in part complement these limitations.

To design more effective anti-COVID platforms, it has been thought that the involvement of cGAMP within viral vaccine vectors enhances immunogenicity [87]. Regarding the immunogenicity potential of adjuvant STING agonists, Liu and co-workers recently designed a novel STING agonist, including an IgG fragment crystallizable region (Fc)-conjugated RBD vaccine combined with CF501, as a vaccine’s adjuvant in a pre-clinical setting. The results demonstrate that it is more potent than Alum- and cGAMP-adjuvanted RBD-Fc [88]. Moreover, CF501 adjuvanted SARS-CoV-2 RBD-Fc (CF501/RBD-Fc) vaccine candidate elicited higher titers of neutralizing Ab (nAb) and durable humoral/cellular immune (T cell) responses accompanied by a lower virus load in the respiratory tract [89, 90].

**Fig. 3:**
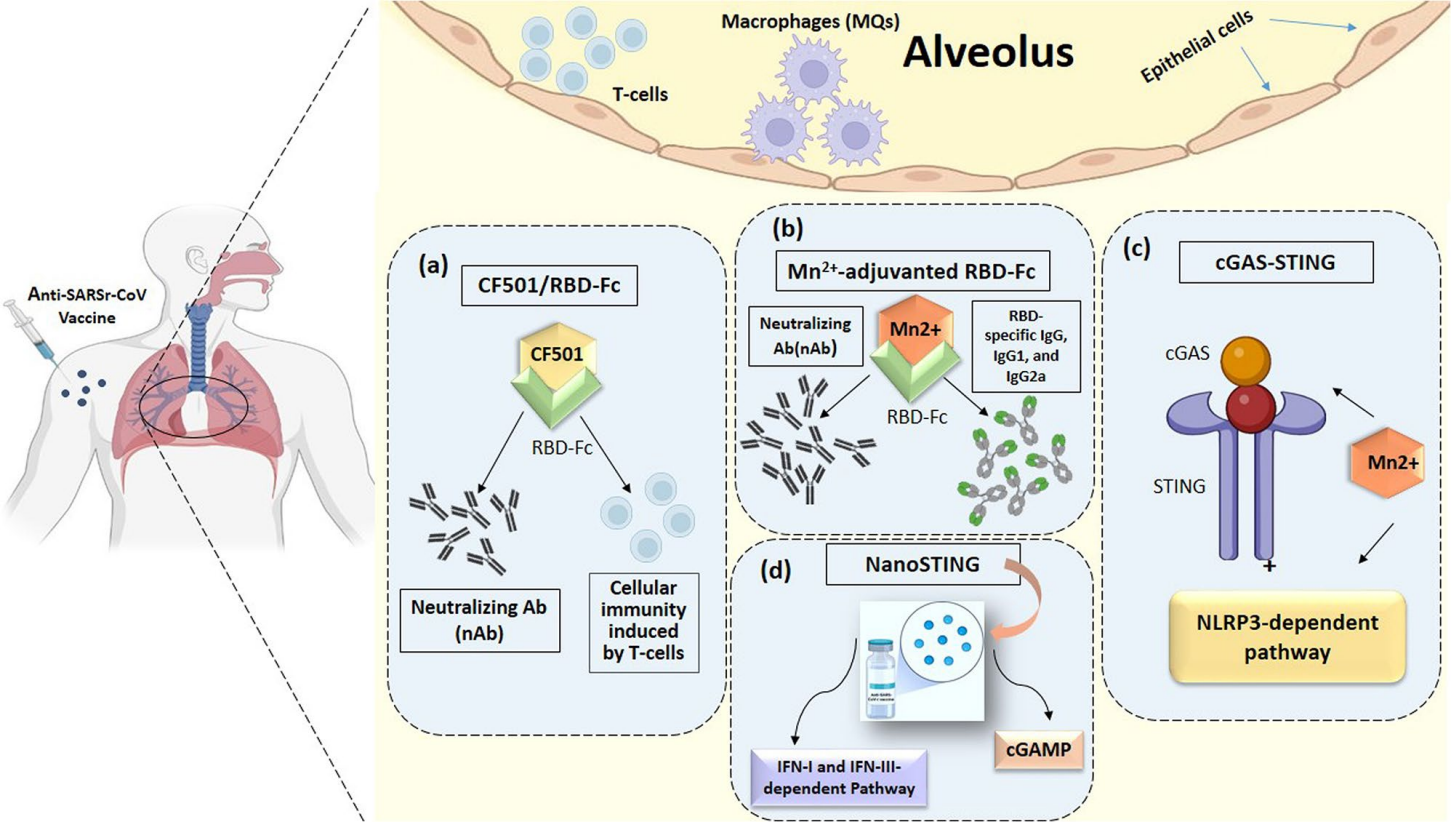
Various STING-agonist adjuvants platforms. All proposed anti-SARS-CoV-2 vaccine adjuvants, including CF501/RBD-Fc (**a**), Mn^2+^- adjuvant RBC-Fc (**b**), cGAS-STING (**c**), and NanoSTING (**d**) are represented in this schematic drawings. *Abbreviation* CF501/RBD-Fc, CF501-adjuvanted SARS-CoV-2 RBD-Fc; Fc, Freund’s complete; IFN-I and -III, Type I and III Interferon; Mn^2+^, Manganese; NanoSTING, Nanoparticle STING agonists; nAb, Neutralizing Antibodies; RBD, receptor binding domain;

Other approaches demonstrated that colloidal manganese (Mn^2+^) salt can be applied as an immune booster and delivery system. This adjuvant provoked humoral/cellular immunity through intramuscular and intranasal routes in a cGAS-STING and NLRP3-dependent manner. Therefore, Mn^2+^ salt is a promising candidate for both cancer and viral infection vaccines [91]. Consistent with this, nanoparticle Mn^2+^ was considered an optimal and potent adjuvant among proposed inactive vaccine ingredients, which could enhance the immunogenicity and potency of the protein-based COVID-19 subunit vaccines, i.e., RBD-Fc, RBD, and S-trimer. The mechanisms of improved protection are attributable to the cGAS-STING pathway, mainly mediated by the Mn^2+^-adjuvanted RBD-Fc, the highest levels of nAb, and RBD-specific IgG, IgG1, and IgG2a when compared with aluminum and MF59 adjuvants (Fig. 3 Table 2) [92].

**Table 2:** STING agonist-adjuvants to augment immunogenicity in vaccines against SARS CoV-2

No.	Adjuvants	Year		Type	Mechanism	Variants/ vaccines		Experimental model	Route	Ref.
1	Mn^2+^	2020		Biological	Via cGAS activation and independent of dsDNA leads to the catalytic synthesis of 2′3′-cGAMP	cGAS -∕ - HeLa cells with wild-type (WT)-cGAS or dZ-cGAS, DNA transfection and DNA virus vaccinia infection		THP-1 cells	-	[8]
2	MnJ (10 µg)	2021		Synthetic	Induces Ab production and CD4+/CD8 + T-cell proliferation and activation via cGAS-STING and NLRP3-ASC pathways, by facilitating Ag uptake	The S1 subunit protein (14–685 aa) of SARSCoV-2		WT mice (C57 BL/6)	IM, IN	[9]
3	Alum	2022		Synthetic	Elevates Ab responses with relatively high RBD-specific IgG, IgG1, and IgG2a responses, and nAb, via cGAS-STING activation	SARS-CoV-2 HKU-001a strain, Delta/ Protein-based COVID-19 vaccines: RBD-Fc		Cell culture and mice SPF female BALB/c	IM	[10]
4	Nanoparticle Mn^2+^ (100 µg)	2022		Synthetic	Induces highest levels of SARS-CoV-2 RBD-specific IgG, IgG1, IgG2a, and nAb via cGAS-STING activation	SARS-CoV-2 HKU-001a, Delta variant/ Protein-based COVID-19: RBD-Fc		Cell culture and mice SPF female BALB/c	IM	[10]
5	MF59	2022		Synthetic	Elevates Ab responses with relatively high RBD-specific IgG, IgG1, and IgG2a, and nAb, via cGAS-STING	SARS-CoV-2 HKU-001a, Delta/ Protein-based COVID-19: RBD-Fc		Cell culture and mice SPF female BALB/c	IM	[10]
6	Small molecule STING agonists (CF501/RBD-Fc, 10 µM in cells, 75 µg in animals	2022		Synthetic	The higher level of phosphorylated STING, TBK1, and IRF3 increases levels of IgG, IgG1 and IgG2a. induces strong cross-nAbs and T-cell responses	SARS-CoV-2 WT, Alpha (B.1.1.7), Beta (B.1.351), Eta (B.1.525), and SARS-CoV/pan-sarbecovirus		THP-1 cells, hACE2-transgenic mice, rabbits, NHPs, e.g., Rhesus Macaques	IM	[11, 12]
7	CF501/RBD-Fc	2022		Synthetic	Increased RBD-specific IgG, nAbs	Omicron (B.1.1.529) and WA1 PsVs/ pan-sarbecovirus		Rhesus Macaques	IM	[13]
8	SARS-CoV-2 RBD, Fc fragment of human IgG,10 µg		2020	Synthetic	Increased RBD-specific and S1-specific Ab (IgG), nAbs		Mutant PsVs and SARSr-CoV/ RBD-Fc-based COVID-19	hACE2 transgenic mice	SC	[14]
9	NanoSTING (60 µg)	2022		Synthetic	Through IFN (-I and -III)-dependent and IFN-independent antiviral pathways, an increase in the concentration of cGAMP		SARS-CoV-2, Alpha, Delta, neutralization-resistant SARS-CoV-2/Omicron	Syrian Golden Haster	IN	[15]
10	An adopting STING agonist: CDN CDG^SF^ (20 µg)	2020		Synthetic	The high IgG titer and a robust S protein-specific T cell response	Inactivated virus, Recombinant RBD protein/peptide, and DNA/RNA vaccines.		J774A.1 (mouse monocyte macrophage cells), mice	SC	[16]

*Abbreviations* Alum, aluminum; CDG, cyclic di-GMP; CDNs, cyclic dinucleotides; Fc, Freund’s complete; IM, intramuscular; IN, intranasal, IFN, interferon; nAb, neutralizing antibodies; hACE2, human Angiotensin converting enzyme2; Mn^2+^, manganese salts; MnJ, manganese jelly; NanoSTING, nanoparticle STING agonists; NHP, nonhuman primates, PsVs, pseudoviruses; RBD, receptor binding domain; SC, subcutaneous; SPF, specific-pathogen-free; WT, wild type

The pan-sarbecovirus (SARS Betacoronavirus) vaccine by design could offer protection against infectious conditions induced by all sarbecovirus strains, especially SARS-CoV-1 and SARS-CoV-2 [88]. Considering newly emerging SARS-CoV-2, Liu et al. designed an RBD-binding Ab, XG014, which can potently neutralize β-coronavirus lineage B (β-CoV-B) with a unique schedule during distinct targeting of conserved epitopes located outside the ACE2 binding.

site by completely blocking RBD in the non-functional “down” conformation, but not in the “up” position [89]. Therefore, this conserved antigenic epitope was supposedly considered a promising.

target for developing ultra-potent vaccines, namely the pan-β-CoV-B therapeutics. Due to enhancing the immunogenicity of the conserved epitopes in RBD, a novel STING agonist adjuvant in the pan-sarbecovirus vaccine, CF501/RBD-Fc, could also induce potent immunogenicity even against SARS-CoV-2 circulating variants [88, 93]. Of note, CF501 facilitates immunity to SARS-COV-2-related RBD that cross-react with other RBDs from different sarbecovirus subtypes [94].

Considering evolving COVID-19 variants, recent advances unveiled the development of vaccines formulated by various adjuvants targeting mucosal immunity to impede SARS-CoV-2 infection. For this purpose, SARS-CoV-2 spike trimer conjugated with a unique adjuvant LP-GMP was designed, consisting of TLR2 and STING agonists by generating potent specific IgG, IgA, and memory T cells (tissue-resident memory T-cells) in both lungs and nasal mucosa in human ACE-2 transgenic (K18-hACE) mice [95]. Another promising STING agonist (c-di-AMP)-based vaccine was designed using monomeric RBD along with CDG^SF^, displaying an enhanced immunogenicity with appreciated neutralizing antibody and Th1-biased immune responses when compared with aluminum hydroxide (Al (OH) 3) [96, 97].

Alternatively, a ternary adjuvant vaccine, comprising of Alum + c-GAMP + poly (I: C) with STING agonist 3, 3′-c-GAMP (c-GAMP) and TLR3 agonist poly (I: C) combined with S1 protein was introduced. This approach represented a significant synergistic impact in favor of eliciting immune responses against live viruses and all variants of concern [98]. More recently, an Mn^2+^-silica nanoplatform (MnO_x_@HMSN) multi-potent vaccine was also developed, amplifying the adjuvant effect of CDN by engaging the STING-I IFN pathway with extended humoral immune response and neutralizing antibodies capability [99]. Therefore, it can be concluded that targeting the cGAS-STING pathway is a promising approach to designing a new generation of RBD/metal/NP-based engineered vaccines against new emerging SARS-CoV-2 variants.

## Conclusion

A growing body of literature has investigated the STING pathway in various RNA/DNA viruses. As shown in Fig. 1, although distinct upstream factors have been reported, they end up in a common pathway for activating the STING signaling pathway. Beyond the favorable effects under various conditions, STING agonists have recently emerged as potential prophylactic/therapeutic agents exploited for patients with SARS-CoV-2 infection. In turn, mounting evidence also supports the fact that the modulation of STING-dependent pathways through various adjuvants provides novel therapeutic targets against immunopathology and immune dysregulation induced by SARS-related coronavirus. Despite multiple engineered carriers and drug-delivery systems, employing both the STING agonists and autophagy modulation in the clinical setting remains to be deciphered in further research.

### Limitations of studies and future prospective

Despite plenty of efforts, data is lacking about the safety and effectiveness of the STING agonists as adjuvants used for SARS-CoV-2 vaccines. On the other hand, according to the appreciated effect of regulated autophagy in developing novel antitumor immunotherapy, designed as a microneedle-assisted vaccination [100], special attention can be also paid in the context of novel antiviral vaccines to combat the threat posed by SARS-related coronavirus.

The compelling evidence highlights that STING agonists can be repurposed as booster adjuvants in SARS-CoV-2 vaccines. This is of particular value for active immunization of susceptible communities, patients with predisposing conditions, senior people, and immunosuppressed recipients against SARS-CoV-2 infection. Considering the magnitude of the well-known detrimental impacts of SARS-CoV-2 pandemics on global health on one hand, and limited data to support this hypothesis that STING agonists can be considered a novel booster adjuvant following the vaccination in the clinical setting, on the other hand, in-depth clinical investigations are greatly recommended.

## Data Availability

No datasets were generated or analysed during the current study.
